# BioServices: a common Python package to access biological Web Services programmatically

**DOI:** 10.1093/bioinformatics/btt547

**Published:** 2013-09-23

**Authors:** Thomas Cokelaer, Dennis Pultz, Lea M. Harder, Jordi Serra-Musach, Julio Saez-Rodriguez

**Affiliations:** ^1^European Molecular Biology Laboratory, European Bioinformatics Institute, Wellcome Trust Genome Campus, Cambridge, CB10 1SD, UK, ^2^Department of Biochemistry and Molecular Biology, University of Southern Denmark, Odense 5230, Denmark, ^3^Translational Research Laboratory, Breast Cancer Unit, Catalan Institute of Oncology (ICO), Bellvitge Institute for Biomedical Research (IDIBELL), Gran via 199, L'Hospitalet del Llobregat, Barcelona 08908, Catalonia, Spain and ^4^Biomedical Research Institute of Girona, Girona 17007, Catalonia, Spain

## Abstract

**Motivation:** Web interfaces provide access to numerous biological databases. Many can be accessed to in a programmatic way thanks to Web Services. Building applications that combine several of them would benefit from a single framework.

**Results:** BioServices is a comprehensive Python framework that provides programmatic access to major bioinformatics Web Services (e.g. KEGG, UniProt, BioModels, ChEMBLdb). Wrapping additional Web Services based either on Representational State Transfer or Simple Object Access Protocol/Web Services Description Language technologies is eased by the usage of object-oriented programming.

**Availability and implementation:** BioServices releases and documentation are available at http://pypi.python.org/pypi/bioservices under a GPL-v3 license.

**Contact:** cokelaer@ebi.ac.uk or bioservices@googlegroups.com

**Supplementary information:** Supplementary data are available at *Bioinformatics* online.

## 1 INTRODUCTION AND MOTIVATION

Many biological databases are accessible on the www (world wide web) via server-side applications that span the entire spectrum of bioinformatics (e.g. genomics, sequence analysis). Although manual requests allow quick retrieval of information, programmatic access via Web Services scales up the number of requests and permits the composition of complex workflows. One strength of Web Services is that client-side applications do not need any intimate knowledge of the database provided by the service itself. Life sciences and bioinformatics have had a fecund production of Web Services in recent years (Bhagat *et al.*, 2010).

Web services integration within a single framework fosters the development of applications. An example based on JAVA is MAPI (Karlsson and Trelles, 2013) that has been a base for developing biomedical applications. Programmatic access to Web Services relies mostly on (i) REST (Representational State Transfer) and (ii) SOAP (Simple Object Access Protocol; www.w3.org/TR/soap). REST has an emphasis on readability: each resource corresponds to a unique URL. There is no need for any external dependency, as operations are carried out via standard Hypertext Transfer Protocol (HTTP) methods (e.g. GET, POST). SOAP uses extensible mark-up language (XML)-based messaging protocol to encode request and response messages using WSDL (Web Services Description Language; www.w3.org/TR/wsdl) to describe the service’s capabilities.

To build applications that integrate several Web Services, one needs to have expertise in (i) HTTP requests, (ii) SOAP protocol, (iii) REST protocol, (iv) XML parsing to consume the XML messages and (v) related bioinformatics fields. Besides, inputs and outputs of the services can be heterogeneous. Consequently, the composition of workflows or design of external applications based on several Web Services can be challenging.

The Python language has many useful features for researchers (Bassi 2007): it is an object-oriented language with a precise and concise syntax and has a versatile set of standard modules. There is a growing and thriving community of scientific developers. An example of a library dedicated to bioinformatics is BioPython (Cock *et al.*, 2009). It provides input/output functions, algorithms and some access to Web Services (e.g. Entrez). However, a dedicated framework to easily integrate bioinformatics Web Services and to provide extensive access to them is missing.

We have, therefore, developed BioServices to provide programmatic access to major bioinformatics Web Services within a single software framework using Python as a glue language. It should alleviate the needs for technical knowledge to develop more complex applications around existing resources.

## 2 APPROACH AND IMPLEMENTATION

**Fig. 1.**
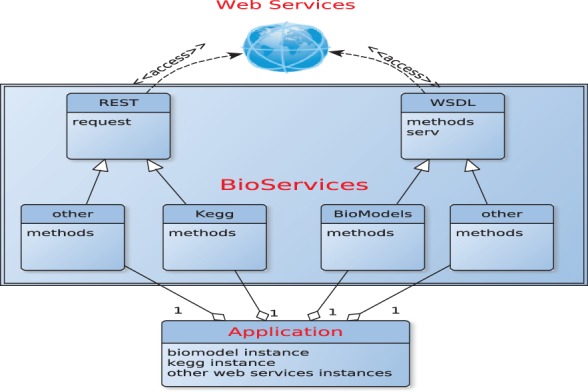
Interaction between external applications and existing Web Services via BioServices. External applications can use BioServices to compose or aggregate several Web Services (see Table 1 for available services)

To bring together various Web Services within BioServices, we first designed two base classes called RESTService and WSDLService so as to ease the wrapping of Web Services. As shown in Figure 1, these two classes are then used by all services available within BioServices. A SOAP/WSDL Web Service can be wrapped concisely as follows:

from bioservices import WSDLService
class AWrapper(WSDLService):
 def __init__(self):
  super(AWrapper, self).__init__(
      "AWrapper", url="validURL?wsdl”)

Similarly, REST services can be exposed concisely (replace *WSDLService* by *RESTService*) as explained in the *Developer Section* of the Supplementary Data. An example of SOAP/WSDL service wrapped within BioServices is BioModels (Li, 2010). Consider the following example:

1 from bioservices import BioModels
2 s = BioModels()
3 s.methods # methods exposed by WSDL
4 s.serv.getAllModelsId()
5 s.getAllModelsId()

All methods exposed by the service are listed in the *methods* attribute (line 3). They can be called directly via the *serv* attribute. For example, all model identifiers can be retrieved (line 4). Methods are then wrapped (line 5) to add robustness and quality.

Web Services currently available in BioServices (see Table 1) can be used independently but they can also be combined. Amongst the various examples provided in the Supplementary Data, a case study demonstrates how to retrieve a protein’s UniProt identifier, its corresponding FASTA sequence, the related Kyoto Encyclopedia of Genes and Genomes (KEGG) pathways, the interactions with other proteins (PSICQUIC) and so forth.

**Table 1.** Web Services accessible from BioServices

ArrayExpress (R)	BioMart (R)	BioModels (W)
ChEBI (W)	ChEMBLdb (R)	EUtils (W)
KEGG (R)	HGNC (R)	Miriam (W)
PDB (R)	PICR (R)	PSICQUIC (R)
QuickGO (R)	Rhea (R)	UniChem (R)
UniProt (R)	NCBIBlast (R)	WikiPathways (W)

*Note*: R stands for REST and W stands for SOAP/WSDL protocol.

Two issues arise when manipulating several services, especially for end-users: (i) heterogeneous data structures are returned and (ii) a plethora of identifiers and keywords are required. Both issues are unfortunately inherent to the diversity of the Web Services used. Although some data structures are commonly used (e.g. XML format), there is still a variety of data structures to deal with. BioServices addresses the first issue by providing extensive documentation and examples. As for the identifiers issue, although BioServices does not provide mapping functions by itself, it gives access to mapping functions from UniProt, KEGG and UniChem (among others). See the online documentation (http://pypi.python.org/pypi/bioservices) for examples.

## 3 CONCLUSION/RESULTS

BioServices provides a comprehensive access to bioinformatics Web Services within a single Python library; the current release (1.1.1) provides access to 18 Web Services (see Table 1). The methodology used to encapsulate Web Services and their functionalities combined with Python allow pipelines (that combine several Web Services) to be implemented concisely. Besides, an extensive online documentation (http://pypi.python.org/pypi/bioservices) should help users and developers to deal with the profusion of identifiers and data structures inherent to the diversity of Web Services available. Releases are available on PyPi (http://pypi.python.org/pypi/bioservices), the official Python repository. Developers can obtain the source code from a public server (https://www.assembla.com/spaces/bioservices/wiki). Besides, bug reports and new feature requests are encouraged (https://www.assembla.com/spaces/bioservices/tickets), and contributors are welcome to join the user and developer community (https://www.assembla.com/spaces/bioservices/wiki). Tests are included with a large coverage to guarantee robustness regarding potential modifications of the Web Services themselves. By covering a wide range of Web Services, BioServices can be used to complement external libraries (e.g. BioPython, Galaxy; see Supplementary Data) and foster the development of new workflows.

*Funding*: Danish Research Councils (to L.M.H. and D.P.), Lundbeck Foundation (to L.M.H.), Foundation Ferran Sunyer i Balaguer (to J.S.M.), Biomedical Research Institute of Girona (to J.S.M.) and EU *BioPreDyn* FP7-KBBE (grant 289434).

*Conflict of Interest*: none declared.

## Supplementary Material

Supplementary Data
