# Supplementary material for: BioServices: a common Python package to access biological Web Services programmatically
Source: Bioinformatics. 2013 Sep 23;29(24):3241–2. doi: 10.1093/bioinformatics/btt547 (PMC3842755; doi:10.1093/bioinformatics/btt547)
Supplement: Supplementary Data [file supp_29_24_3241__index.html]

BioServices: a Common Python Package to Access Biological Web Services Programmatically — BioServices: a common Python package to access biological Web Services programmatically — BioServices: a common Python package to access biological Web Services programmatically — Supplementary Data 

# BioServices: a common Python package to access biological Web Services programmatically

## Supplementary Data

files

**Files in this Data Supplement:**

- Supplementary Data - pdf file
