# Supplementary material for: BioServices: a common Python package to access biological Web Services programmatically
Source: Bioinformatics. 2013 Sep 23;29(24):3241–2. doi: 10.1093/bioinformatics/btt547 (PMC3842755; doi:10.1093/bioinformatics/btt547)
Supplement: Supplementary Data [file supp_btt547_cokelaer_bioservices_supp.pdf]

Supplementary Material - BioServices(1.1.1)

August 2013

Contents

Page

1 Introduction and Installation . . . 2
2 Tutorial 1: Protein test case study . . . 3
3 Tutorial 2: Converting compound identifiers . . . 9
4 Applications: Combining BioServices and PyMol . . . 12
5 Applications: Combining BioServices and BioPython . . . 14
6 Applications: Combining BioServices and Galaxy . . . 15
7 Short Developer Guide . . . 17
8 More information . . . 19

List of Figures

1 Visualisation of the 3D structure of ZAP70 shown within PyMOL software. The input PDB files was fetched with BioServices. . . . 13

Notes

Note 1: This supplementary data is a subset of the on-line documentation version 1.1.1. See the full up-to-date documentation on-line on Pypi (the Python package repository) – http://pythonhosted.org/bioservices/

Note 2: Contributions to implement new wrappers are more than welcome. See BioServices wiki to join the development, and the *Developer Guide* on how to implement new wrappers.

Note 3: The examples have been tested with BioServices version 1.1.1 and Python 2.7.1

---

1 Introduction and Installation

BioServices software has a thorough documentation available on Pypi (the Python package repository) that is up-to-date. The on-line documentation provides a User Guide as well as a Reference Guide. All classes and functions are documented and test coverage is around 80%.

The source code of **BioServices** is also available on PyPi. The following command should install **BioServices** and its dependencies automatically provided you have **PIP** installed on your system:

```
sudo pip install bioservices
```

If not, please see the external pip installation page. If **PIP** fails to install bioservices you may want to try easy_install tool instead:

```
sudo easy_install bioservices
```

In the first section of this supplementary data (Section 2, we reproduce part of the tutorial available in the on-line **BioServices** documentation. The tutorial demonstrates how to use BioServices classes to obtain information about a protein using different Web services available through BioServices. The Section 4 provides an example that combines BioServices with an external application called PyMOl that demonstrate how to combine application with BioServices. Similarly, the following two sections show how to use BioServices and BioPython together and how to write a plugin for Galaxy. The last section 7 is for developers (or users) who want to implement a new class dedicated to a given Web Services that is not available in BioServices (based on either the REST or WSDL protocol).

2 Tutorial 1: Protein test case study

Application: retrieving information about a given protein

This section uses BioServices to demonstrate the interest of combining several services together within a single framework using the Python language as a glue language.

In this tutorial we are interested in using **BioServices** to obtain information about a specific protein. Let us focus on the protein known as ZAP70 (homo sapiens).

2.1 Get a unique identifier and gene names from a name

Given the gene name of a protein, we first want to obtain its unique Uniprot identifier. Using the **UniProt** class provided in BioServices, we can obtain the unique accession number of ZAP70, which may be useful later on. Let us first create an instance of **UniProt** service and use the the `UniProt.search()` method:

```
>>> from bioservices import *
>>> u = UniProt(verbose=False)
>>> res = u.search("ZAP70_HUMAN") # could be lower case
```

The default format of the returned answer is in tabulated format. Other formats such as HTML, XML could be used using the *format* argument. Let us now print the results returned by the *search* method.

```
>>> print(res)
Entry   Entry name  Status   Protein names    Gene names  Organism     Length
P43403  ZAP70_HUMAN reviewed    Tyrosine-protein kinase ZAP-70 ZAP70 SRK Homo ...
```

It is better, but let us simplify even further. In **BioServices**, the output of the tabulated format contains several columns but we can select only a subset such as the Entry (accession number) and the gene names, which are coded as “id” and “genes” in uniprot database:

```
>>> res = u.search("ZAP70_HUMAN", format="tab", columns="id,genes")
>>> print(res)
Entry   Gene names
P43403  ZAP70 SRK
```

So here we got the Entry P43403, which is the unique identifier we were looking for. In this case, it was easy because the input name is a gene name itself. In some other cases, one may need to introspect the description or protein names instead of the gene names only.

2.2 Getting the fasta sequence

It is then straightforward to obtain the FASTA sequence of ZAP70 using another method from the UniProt class called `searchUniProtId()`:

```
>>> sequence = u.searchUniProtId("P43403", "fasta")
>>> print(sequence)
>sp|P43403|ZAP70_HUMAN Tyrosine-protein kinase ZAP-70 OS=Homo sapiens GN=ZAP70 ...
MPDPAAHLPFFYGSISRAEAEEHLKLAGMADGLFLLRQCLRSLGGYVLSLVHDVRFHHFP
IERQLNGTYAIAGGKAHCGPAELCEFYSRDPDGLPCNLRKPCNRPSGLEPQPGVFDCLRD
AMVRDYVRQTWKLEGEALEQAIISQAPQVEKLIATTAHERMPWYHSSLTREEAERKLYSG
AQTDGKFLLRPRKEQGTYALSLIYGKTVYHYLISQDKAGKYCIPEGTKFDTLWQLVEYLK
```

```
LKADGLIYCLKEACPNSSASNASGAAAPTLPAHPSTLTHPQRRIDTLNSDGYTPEPARIT
SPDKPRPMPMDTSVYESPYSDPEELKDKKLFLKRDNLLIADIELGCGNFGSVRQGVYRMR
KKQIDVAIKVLKQGTEKADTEEMMREAQIMHQLDNPYIVRLIGVCQAEALMLVMEMAGGG
PLHKFLVGKREEIPVSNVAELLHQVSMGMKYLEEKNFVHRDLAARNVLLVNRHYAKISDF
GLSKALGADDSYYTARSAGKWPLKWYAPECINFRKFSSRSDVWSYGVTMWEALSYGQKPY
KKMKGPEVMAFIEQGKRMECPPECPPELYALMSDCWIYKWEDRPDFLTVEQRMRACYYSL
ASKVEGPPGSTQKAEAACA
```

Note: There are many services that provides access to the FASTA sequence. We chose **UniProt** but you could use another service such as the Entrez utilities (EUtils class in BioServices).

2.3 Using BLAST on the sequence

You can then analyse this sequence with your favourite tool. As an example, within **BioServices** you can use `NCIBlast` class but first let us extract the sequence itself (without the header) using some standard Python code:

```
sequence = sequence.split("\n", 1)[1].strip("\n")
```

then we create a NCBIblast instance and run the analysis by specifying the blast variant (here *blastp*),

```
>>> s = NCBIblast(verbose=False)
>>> jobid = s.run(program="blastp", sequence=sequence, stype="protein", \
...     database="uniprotkb", email="youremail@domain")
>>> print s.getResult(jobid, "out")[0:1000]
BLASTP 2.2.26 [Sep-21-2011]


Reference: Altschul, Stephen F., Thomas L. Madden, Alejandro A. Schaffer,
Jinghui Zhang, Zheng Zhang, Webb Miller, and David J. Lipman (1997),
"Gapped BLAST and PSI-BLAST: a new generation of protein database search
programs",  Nucleic Acids Res. 25:3389-3402.

Query= EMBOSS_001
         (619 letters)

Database: uniprotkb
           32,727,302 sequences; 10,543,978,207 total letters

Searching..................................................done



                                                                 Score    E
Sequences producing significant alignments:                      (bits) Value

SP:ZAP70_HUMAN P43403 Tyrosine-protein kinase ZAP-70 OS=Homo sap...  1279   0.0
TR:H2QIE3_PANTR H2QIE3 Tyrosine-protein kinase OS=Pan troglodyte...  1278   0.0
TR:G3QGN8_GORGO G3QGN8 Tyrosine-protein kinase OS=Gorilla gorill...  1278   0.0
TR:G1QLX3_NOMLE G1QLX3 Tyrosine-protein kinase OS=Nomascus leuco...  1249   0.0
TR:F6SWY7_CALJA F6SWY7 Tyrosin
```

The last command waits for the job to be finished before printing the results, which may takes a few minutes depending on the NCBI server. We could look at the beginning of the reported results and select only HUMAN sequences to see that the best sequence found correspond indeed to ZAP70_HUMAN (as expected!):

```
>>> [x for x in s.getResult(jobid, "out").split("\n") if "HUMAN" in x]
['SP:ZAP70_HUMAN P43403 Tyrosine-protein kinase ZAP-70 OS=Homo sap... 1279 0.0  ',
 'SP:KSYK_HUMAN P43405 Tyrosine-protein kinase SYK OS=Homo sapiens...  691 0.0 ',
 'TR:A8K4G2_HUMAN A8K4G2 Tyrosine-protein kinase OS=Homo sapiens P...  691 0.0 ',
...
```

2.4 Searching for relevant pathways

The KEGG service provides pathways, so let us try to find pathways that contain our targeted protein. First we need to know the KEGG Id that corresponds to ZAP70. We can use the **find** method form KEGG service:

```
>>> from bioservices import Kegg
>>> k = Kegg(verbose=False)
>>> k.find("hsa", "zap70")  # "hsa" stands for homo sapiens
hsa:7535 ZAP70, SRK, STCD, STD, TZK, ZAP-70; zeta-chain (TCR) associated protein
 kinase  70kDa (EC:2.7.10.2); K07360 tyrosine-protein kinase ZAP-70 [EC:2.7.10.2
```

Now, let us get the pathways that contains this ID:

```
>>> k.get_pathway_by_gene("7535", "hsa")
{'hsa04064': ' NF-kappa B signaling pathway',
'hsa04650': 'Natural killer cell mediated cytotoxicity',
 'hsa04660': 'T cell receptor signaling pathway',
 'hsa05340': 'Primary immunodeficiency'}
```

We can look at the first pathway in a browser (highlighting the ZAP70 node):

```
>>> k.show_pathway("hsa04064", keggid={"7535": "red"})
```

2.5 Searching for binary Interactions

Another interesting Web Service available within BioServices is **PSICQUIC**. This is actually a portal to 25 databases that provide protein interactions. As an example, we can search for interactions that involve the ZAP70 protein within the **mint** database. The code is as follows:

```
>>> from bioservices import PSICQUIC
>>> s = PSICQUIC(verbose=False)
>>> data = s.query("mint", "ZAP70 AND species:9606")
```

where 9606 is the taxonomy identifier for the *homo sapiens* specy. We can check the number of interactions involved is 34:

```
>>> len(data)
34
```

We could also figure out how many interactions could be found in each database for this particular query:

```
>>> s.getInteractionCounter("zap70 AND species:9606")
{'apid': 82,
 'bar': 0,
 'bind': 4,
 'bindingdb': 29,
 'biogrid': 73,
 'chembl': 161,
 'dip': 0,
```

```
 'i2d-imex': 0,
 'innatedb': 13,
 'innatedb-imex': 0,
 'intact': 11,
 'interoporc': 0,
 'irefindex': 273,
 'matrixdb': 0,
 'mbinfo': 0,
 'mint': 34,
 'molcon': 0,
 'mpidb': 0,
 'reactome': 0,
 'reactome-fis': 134,
 'spike': 47,
 'string': 319,
 'topfind': 0,
 'uniprot': 0}
```

We see for instance that the **mint** database has 34 interactions (as already found earlier). Coming back to the data found in the mint database only, we can look at the first entry:

```
>>> for x in data[0]: print(x)
uniprotkb:P15498
uniprotkb:P43403
-
-
uniprotkb:VAV1(gene name)|uniprotkb:VAV(gene name synonym)
uniprotkb:ZAP70(gene name)|uniprotkb:SRK(gene name synonym)|uniprotkb:70 kDa
zeta-associated protein(gene name synonym)|uniprotkb:Syk-related tyrosine
kinase(gene name synonym)
psi-mi:"MI:0019"(coimmunoprecipitation)
-
pubmed:9151714
taxid:9606(Homo sapiens)
taxid:9606(Homo sapiens)
psi-mi:"MI:0914"(association)
psi-mi:"MI:0471"(mint)
mint:MINT-8035351
mint-score:0.28(free-text)|homomint-score:0.28(free-text)'intact-miscore:0.60']
```

The First two elements are the entries for specy A and B. The last element is the score. The 11th element is the type of interaction and so on.

What could be useful is to convert these elements into uniprot ID only. With mint database it is irrelevant (already in uniprot ID format) for this particular entry but with other DBs or entries, it may be useful (e.g., biogrid).

If the following example do no work with *biogrid*, it may be that the service if inactive and you may try to replace *Biogrid* with another service such as *mint*, *string*, ... BioServices provides such a function called `convert()`:

```
>>> data = s.query("biogrid", "ZAP70 AND species:9606")
>>> data2 = s.convert(data, "biogrid")
```

convert method converts all entries from data into uniprot ID. If this is not possible, the entry is removed. The **query** and **convert** works on a single database but you we could query all or a subset of all databases using the queryAll and convertAll functions:

```
>>> data = s.queryAll("ZAP70 AND species:9606", databases=["mint", "biogrid"])
>>> data2 = s.convertAll(data)
```

However, extra cleaning is required to remove entries that are not relevant (no match to uniprot ID, redundant, not a protein,

self interactions, ...). In order to ease this task, the psicquic.AppsPPI class is very useful.

```
from bioservices import psicquic
s = psicquic.AppsPPI()
s.queryAll("ZAP70 AND species:9606", databases=["mint", "biogrid", "intact",
   "reactome-fis"])
s.summary()
s.show_pie()
```

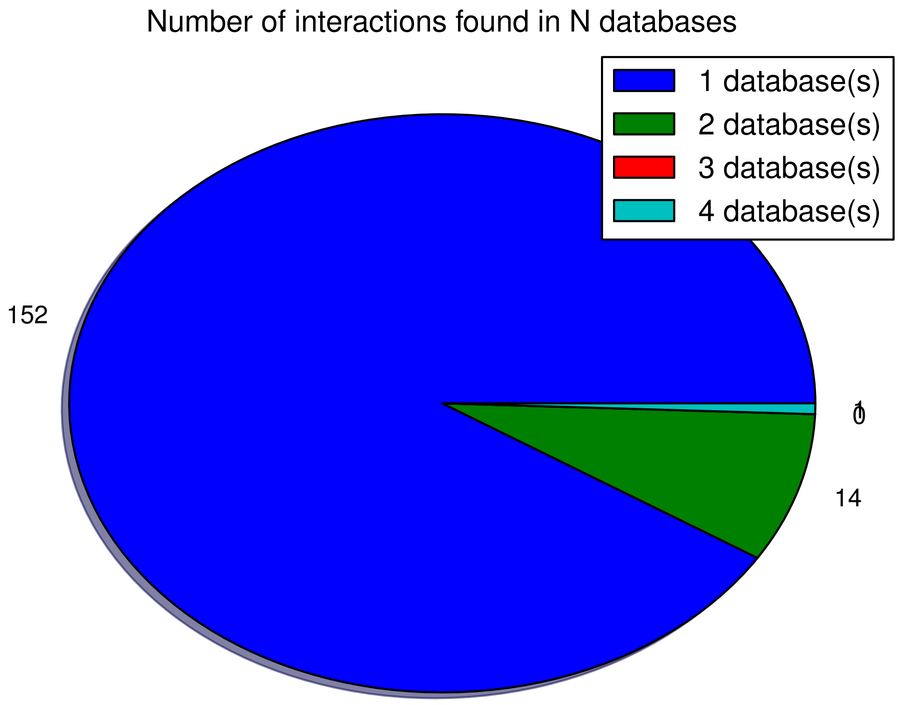

The summary function prints a useful summary about the number of found interactions and overlap between databases:

```
>>> s.summary()
Found 8 interactions within intact database
Found 124 interactions within reactome-fis database
Found 19 interactions within mint database
Found 67 interactions within biogrid database
-------------
Found 152 interactions in 1 common databases
Found 14 interactions in 2 common databases
Found 0 interactions in 3 common databases
Found 1 interactions in 4 common databases
```

Finally, you can obtain the relations that were found in the 4 databases (only 1 here):

```
>>> s.relevant_interactions[4]
[['LCK_HUMAN', 'ZAP70_HUMAN']]
```

2.6 What's next ?

There are lots of other Web Services wrapped within BioServices that could be useful to retrieve more information about the protein ZAP70. An example is the WikiPathway (see `Wikipathway`) to retrieve even more pathways. Another example is the BioMart portal. You could use it to retrieve pathways from REACTOME (see `BioMart`). You can also retrieve targets from ChEMBL given the uniprot ID (get_target_by_uniprotId("P43403")) and so on.

The full documentation of BioServices (on PyPi repository) provides more tutorials and examples covering other aspects of Web Services accessible from **BioServices**.

3 Tutorial 2: Converting compound identifiers

Application: retrieving information about a compound

This section uses BioServices to demonstrate the interest of combining several services together within a single framework using the Python language as a glue language

Retrieve a compound identifier from KEGG, ChEBI and ChEMBL

Let us look at a compound called **Geldanamycin** that inhibits Hsp90. Let us search for information about that compound in several databases and manipulate the different identifiers.

First, let us retrieve information on KEGG database:

```
>>> from bioservices import *
>>> k = Kegg(verbose=False)
```

KEGG compounds have links to other databases. It is not systematic but the ChEBI database is often referenced. So we will want to convert the KEGG identifer to a ChEBI identifier. Later, we can convert a ChEBI to a ChEMBL identifier using another Web Service such as UniChem.

We can get a mapping dictionary from the KEGG compound to ChEBI as follows:

```
>>> map_kegg_chebi = k.conv("chebi", "compound")
>>> len(map_kegg_chebi)
6896

>>> print(k.find("compound", "geldanamycin"))
cpd:C11222  Geldanamycin
cpd:C15823  Progeldanamycin
```

Let us look at the first one (Kegg id cpd:C11222). We can get lots of information from KEGG already by using:

```
>>> print(k.get("C11222"))
```

Form which, there is a link to other databases in particular ChEBI (ChEBI:5292). We could use the mapping dictionary created above:

```
>>> map_kegg_chebi['cpd:C11222']
'chebi:5292'
```

Unfortunately, there is no mapping function from KEGG to ChEMBL in KEGG Web Service.

However, BioServices provides access to the `bioservices.unichem` service. This service provides a useful mapping function from kegg to chembl:

```
>>> uni = UniChem()
>>> mapping = uni.get_mapping("kegg_ligand", "chembl")
>>> mapping['C11222']
'CHEMBL278315'
```

For sanity check, let us see that the ChEBI is indeed 5292 as given within the KEGG database:

```
>>> uni = UniChem()
>>> mapping = uni.get_mapping("kegg_ligand", "chebi")
```

```
>>> mapping['C11222']
'5292'
```

(2) In order to convert KEGG gene names into uniprot gene name, we can also use the UniProt web service from BioServices as follows:

```
>>> from bioservices import *
>>> u = UniProt()
>>> u.mapping(fr='ID', to='KEGG_ID', format='tab', query="ZAP70_HUMAN")

['From:ID', 'To:KEGG_ID', 'P43403', 'hsa:7535']
```

You can get accession number or protein name identifier from the KEGG identifier as follows:

```
>>> u.mapping(fr='KEGG_ID', to='ID', format='tab', query='hsa:7535')
'ZAP70_HUMAN'
>>> u.mapping(fr='KEGG_ID', to='ACC', format='tab', query='hsa:7535')
'P43403'
```

3.1 Mapping identifiers

There are quite a few functions from different Web Services that can help to map identifiers from one database to the other. This tutorial presents some of them.

Convert from KEGG ID to ChEBI (compound)

```
>>> from bioservices import *
>>> k = Kegg(verbose=False)
>>> map_kegg_chebi = k.conv("chebi", "compound")
>>> map_kegg_chebi['cpd:C11222']
'chebi:5292'
```

you could also use `bioesrvices.unichem.UniChem` (see below).

Convert from KEGG ID to ChEMBL (compound)

```
>>> from bioservices import UniChem
>>> uni = UniChem()
>>> mapping = uni.get_mapping("kegg_ligand", "chembl")
>>> mapping["C11222"]
'CHEMBL278315'
```

convert from KEGG ID to UniProt and vice versa (gene)

In order to convert KEGG gene names into uniprot gene name, we can also use the UniProt web service from BioServices as follows:

```
>>> from bioservices import *
>>> u = UniProt()
>>> u.mapping(fr='ID', to='KEGG_ID', format='tab', query="ZAP70_HUMAN")
['From:ID', 'To:KEGG_ID', 'ZAP70_HUMAN', 'hsa:7535']
```

You can get accession number or protein name identifier from the KEGG identifier as follows:

```
>>> u.mapping(fr='KEGG_ID', to='ID', format='tab', query='hsa:7535')
'ZAP70_HUMAN'
>>> u.mapping(fr='KEGG_ID', to='ACC', format='tab', query='hsa:7535')
'P43403'
```

One can also use the `bioservices.kegg.Kegg.conv()` method:

```
>>> k = Kegg()
>>> mapping_kegg_uniprot = k.conv("hsa", "uniprot")
```

The full documentation of BioServices (on PyPi repository) provides more tutorials and examples covering other aspects of Web Services accessible from **BioServices**.

4 Applications: Combining BioServices and PyMol

This section shows how to use BioServices as an intermediate tool to fetch data thanks to web service and then to use this data as an input to an external software (in Python).

We will use PyMOL http://www.pymol.org/, which is a an open-source molecular visualization system. This software is not part of BioServices we do provide specific instructions for the installation. The Reader should refer to PyMOL web site instead (URLs above). However, here is what we did on a Fedora distribution:

```
bunzip pymol-v1.6alpha1.tar.bz2
tar xvf pymol-v1.6alpha1.tar
cd pymol
python setup.py install
```

So, how to link BioServices and PyMOL ? PyMOL can read PDB file that contains the 3D structure of a molecule. Using the same UniProt ID as the one used in the first section (P43403), we can use `bioservices.uniprot.UniProt` to get its accession number (P43403) and its PDB identifier as shown in the example below. Then, we use `bioservices.pdb.PDB` to get the 3D structure in PDB format.

```
import __main__
__main__.pymol_argv = [ 'pymol', '-qc'] # Quiet and no GUI

import os
if os.path.isfile("bioservices_pdb.png"):
    os.remove("bioservices_pdb.png")

# BioServices 1: obtain the PDB ID from a given uniprot ID (P43403 i.e. ZAP70)
from bioservices import *
print("Retrieving PDB ID")
u = UniProt(verbose=False)
res = u.mapping(fr="ID", to="PDB_ID", query="P43403", format="tab")
pdb_id = res[3]    # "1FBV"

# BioServices 2: Download the PDB file from the PDB Web Service
print("Fetching PDB file")
p = pdb.PDB()
res = p.getFile(pdb_id, "pdb")

# General: save the fetched file in a temporary file
import tempfile
fh = tempfile.NamedTemporaryFile()
fh.write(res)
sname = fh.name

# THIS IS NOT BIOSERVICES ANYMORE but PYMOL
import pymol
pymol.finish_launching()
pymol.cmd.load(sname)
pymol.cmd.png("bioservices_pdb.png", width="15cm", height="15cm", dpi=140)
#pymol.cmd.png("my_image.png")
# Get out!
pymol.cmd.quit()
```

The script above uses PyMOL in a script manner to save the 3D graphical representation of the protein (here below) but you could also use PyMOL in an interactive mode.

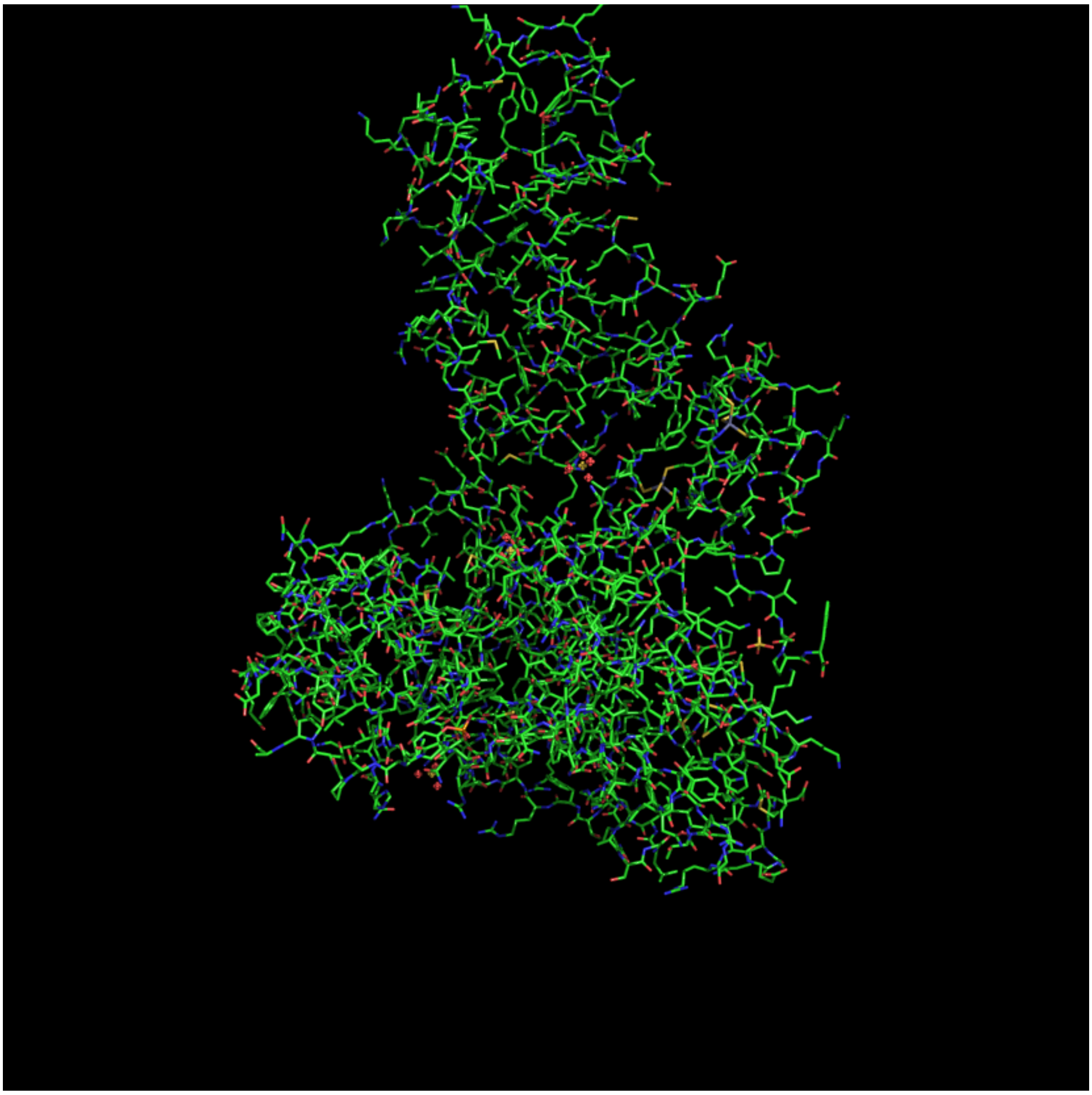

Figure 1: Visualisation of the 3D structure of ZAP70 shown within PyMOL software. The input PDB files was fetched with BioServices.

5 Applications: Combining BioServices and BioPython

http://biopython.org/DIST/docs/tutorial/Tutorial.html#chapter:Bio.AlignIO

BioPython provides many tools for IO, algorithms and some access to Web services. We believe that BioServices can help BioPython users to get on-line resources easily and analyse them with BioPython.

This example shows how (i) to use BioServices to retrieve FASTA files and (ii) BioPython to play with the sequences. The analysis part using BioPython is very simple but serve as an example on how to bridge the 2 packages.

Note: You need to install BioPython and we let the reader refer to the BioPython main page http://biopython.org

---

First, let us retrieve two FASTA sequences and save them in 2 files:

```
from bioservices import UniProt
u = UniProt()
akt1 =u.searchUniProtId("P31749","fasta")
akt2 =u.searchUniProtId("P31751","fasta")

fh =open("akt1.fasta","w")
fh.write(akt1)
fh.close()

fh =open("akt2.fasta","w")
fh.write(akt2)
fh.close()
```

Now, on the BioPython side, we read the 2 sequences and introspect them:

```
>>>  from Bio import AlignIO
>>>  record1=SeqIO.read("akt1.fasta","fasta")
>>> record2=SeqIO.read("akt2.fasta","fasta")
>>> record1+= "-"
# this is to have 2 sequences of same length as requested by the followingfunction

>>> alignment =AlignIO.MultipleSeqAlignment([])
>>>alignment.append(record1)
>>>alignment.append(record2)

>>> forrecord in alignment:
>>> print(description)
sp|P31749|AKT1_HUMAN RAC-alphaserine/threonine-protein kinase OS=Homo sapiens...
sp|P31751|AKT2_HUMAN RAC-betaserine/threonine-protein kinase OS=Homo sapiens...
```

You are ready to play with more BioPython multiple alignment tools. Please consult BioPython documentation for further analysis of the FASTA sequence.

6 Applications: Combining BioServices and Galaxy

Galaxy URL: http://wiki.galaxyproject.org/FrontPage

Galaxy is an open, web-based platform for accessible, reproducible, and transparent computational biomedical research. It provides worflows and plugins to many web resources.

This tutorial shows how to link bioservices and galaxy. Our tutorial will provide a plugin to Galaxy so that a user can retrieve a FASTA file via BioServices and the wrapping of UniProt Web Services.

We assume that you installed Galaxy on your system via the source code:

```
hg clone https://bitbucket.org/galaxy/galaxy-dist/
cd galaxy-dist
hg update stable
```

The tree directory should therefore contains a directory called **tools/** and in the main directory, an XML file called **conf_tools.py**

We will first create a plugin for bioservices. This is done by adding a directory called bioservices in ./tools:

```
mkdir tools/bioservices
```

In this directory, we will create two files called **uniprot.py** that will contain the actual code that calls bioservices and a second XML file that will allows us to design the plugin layout.

Let us start with the plugin. It is very simple since only the UniProt Entry is required. The output will simply be the FASTA file that would have been fetched.

The XML file is:

```
<tool id="bioservices_uniprot" name="Get FASTA" version="1.1.1">
  <description>from UniProt via Bioservices</description>
  <requirements>
    <requirement type="package">bioservices</requirement>
  </requirements>
  <command interpreter="python">uniprot.py $uniprot_id $output</command>
  <inputs>
    <param name="uniprot_id" type="text" label="UniProt ID"
        size="40" help="Provide a valid UniProt Entry (e.g. P43403) "/>
  </inputs>
  <outputs>
    <data format="fasta" name="output" />
  </outputs>
  <help>
Fetch a FASTA file using UniProt via BioServices.
Simply provide a valid Uniprot Entry (e.g., P43403)
  </help>
</tool>
```

The python code will take as an input the UniProt ID and create a file that contains the FASTA data:

```
import sys

def __main__():
    ids = sys.argv[1]
    filename = sys.argv[2]
    # TODO: check the validity and format ?
    try:
        from  bioservices import UniProt
        u = UniProt(verbose=False)
        u.debugLevel = "ERROR"
```

```
    except ImportError:
        print("Could not import bioservoces ? Check that it is installed. '")

    try:
        fasta = u.searchUniProtId(ids, "fasta")
    except:
        print("An error occured while fetching the FASTA file from uniprot")

        fh = open(filename, "w")
        fh.write(fasta)
        fh.close()

if __name__ =='__main__':
    __main__()
```

finally, you need to make Galaxy aware of this new plugin. this is done in the file called conf_tool.xml. Add bioservices plugin. The beginning of the file should look like:

```
<?xml version="1.0"?>
 <toolbox>
   <section name="Get Data" id="getext">
     <tool file="bioservices/uniprot.xml"/>
     <tool file="data_source/upload.xml"/>
...
```

Once done. start you galaxy server. The following image show the outcome: in the left hand side, you can select the bioservices plugin. Then, in the center, you can enter a uniprot entry. Press the execute button and the new file should appear in the right hand side. From there you can use Galaxy other tools to analyse the file.

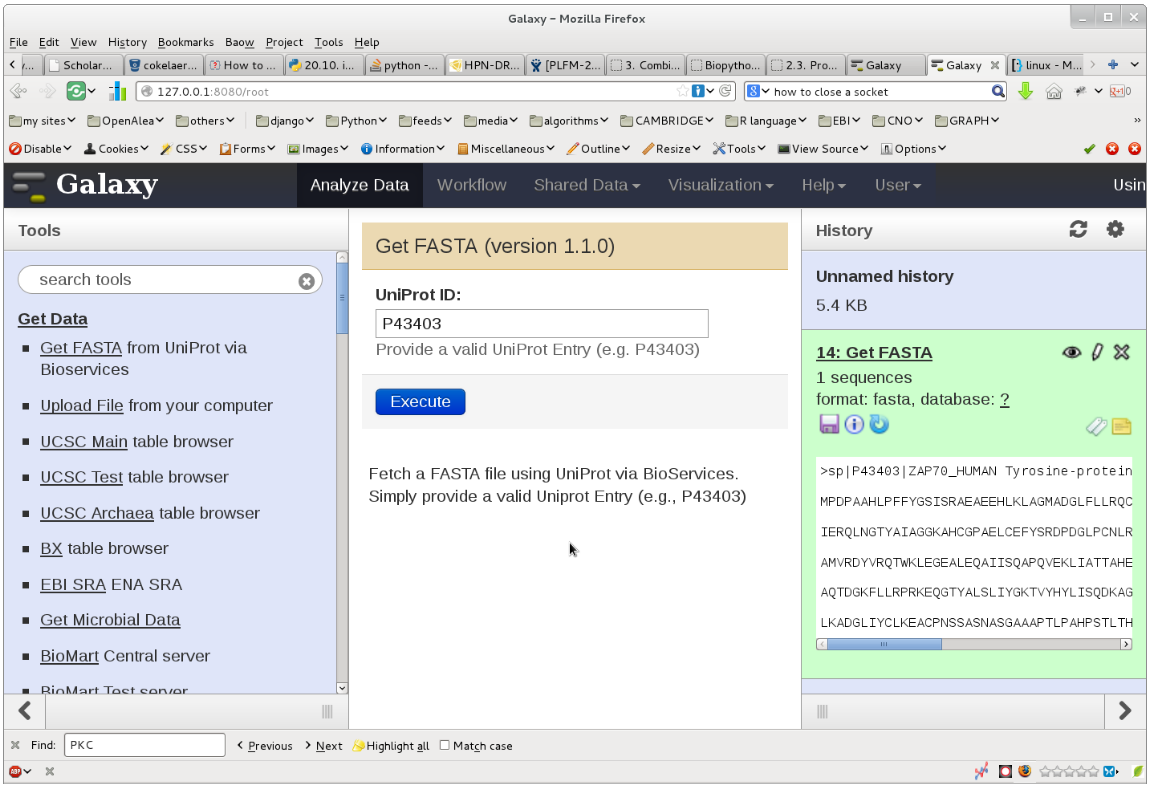

This example shows that it is possible to link Galaxy and BioServices to access to various Web Services that are available through Bioservices. The code related to BioServices is actually made of 3 lines showing that it should be feasible for anyone to include much more functionalities in Galaxy.

7 Short Developer Guide

If a Web Service is not available within **BioServices**, it is still feasible in a few lines of code to access programmatically to these services. The following sections illustrate how to implement dedicated classes to a Web Service based on the REST or WSDL protocol.

7.1 Creating a service class (REST case)

In the REST case, let us take the example of BioMart (which is now in BioServices as well but for this illustration let us suppose it is not). In order to access to the BioMart REST service, you need to know the main URL (provided on their web site) and type:

```
>>> from bioservices import RESTService
>>> s = RESTService("BioMart" ,"http://www.biomart.org/biomart/martservice")
```

The first parameter is compulsary but can be any word. You can retrieve the base URL by typing:

```
>>> s.url
'http://www.biomart.org/biomart/martservice'
```

You are ready to send request to the Web Service. BioServices will not replace the Web Service itself, so you need to figure out from the Web Service documentation itself what are the valid request. As an example let us send a request to retrieve the registry information for instance (see http://www.biomart.org.martservice.html for other valid requests):

```
>>> s.request("?type=registry")
<bioservices.xmltools.easyXML at 0x3b7a4d0>
```

The request method available from RESTService class concatenates the URL and the parameter provided so it requests the "http://www.biomart.org.biomart/martservice?type=registry" URL.

The output is an XML document. If a service returns an XML, we systematically convert it to an XML structure using the BeautilSoup package so as to have a uniform user interface.

As a developer, you should ease the life of the user by wrapping up the previous command. An example of a BioMart class with a unique method dedicated to the registry would look like:

```
>>> class BioMart(RESTService):
...     def __init__(self):
...         url = "http://www.biomart.org/biomart/martservice"
...         super(BioMart, self).__init__("BioMart", url=url)
...     def registry(self):
...         ret = self.request("?type=registry")
...         return ret
```

and you would use it as follows:

```
>>> s = BioMart()
>>> s.registry()
<bioservices.xmltools.easyXML at 0x3b7a4d0>
```

Note that the BioMart is now accessible from BioServices

7.2 Creating a service class (WSDL case)

What about WSDL protocol. For the developer it is almost the same as we will show using the Ontology Lookup service, which provides a WSDL service. In order to easily access this service, we can use the `WSDLService` class as follows:

```
>>> from bioservices import WSDLService
>>> url = "http://www.ebi.ac.uk/ontology-lookup/OntologyQuery.wsdl"
>>> ols = WSDLService("OLS", url)
```

You can now see which methods are available:

```
>>> ols.methods
```

and call one (e.g., getVersion) using the `bioservices.services.WSDLService.serv` attribute:

```
>>> ols.serv.getVersion()
```

You can then look at something more complex and extract relevant information:

```
>>> [x.value for x in ols.serv.getOntologyNames()[0]]
```

Of course, you can add new methods to ease the access to any functionalities:

```
>>> ols.getOnlogyNames() # returns the values
```

Similarly to the previous case using REST, you can wrap this example into a proper class to make the interface simpler, more intuitive, more robust.

8 More information

For more information about BioServices, please visit https://pypi.python.org/pypi/bioservices and consult the entire documentation (user guide, tutorial, developer and reference guides) at http://pythonhosted.org/bioservices/.
